# Association of Elbow Flexor MRI Fat Fraction With Loss of Hand-to-Mouth Movement in Patients With Duchenne Muscular Dystrophy

**DOI:** 10.1212/WNL.0000000000012724

**Published:** 2021-10-26

**Authors:** Karin J. Naarding, Menno van der Holst, Erik W. van Zwet, Nienke M. van de Velde, Imelda J.M. de Groot, Jan J.G.M. Verschuuren, Hermien E. Kan, Erik H. Niks

**Affiliations:** From the Department of Neurology (K.J.N., N.M.v.d.V., J.J.G.M.V., E.H.N.), Duchenne Center Netherlands (K.J.N., M.v.d.H., N.M.v.d.V., I.J.M.d.G., J.J.G.M.V., H.E.K., E.H.N.)Department of Orthopedics, Rehabilitation and Physiotherapy (M.v.d.H.), and Department of Biomedical Data Sciences (E.W.v.Z.), Leiden University Medical Center; and Department of Rehabilitation (I.J.M.d.G.), Radboud University Medical Center, Nijmegen, the Netherlands.

## Abstract

**Background and Objectives:**

To study the potential of quantitative MRI (qMRI) fat fraction (FF) as a biomarker in nonambulant patients with Duchenne muscular dystrophy (DMD), we assessed the additive predictive value of elbow flexor FF to age at loss of hand-to-mouth movement.

**Methods:**

Nonambulant patients with DMD (age ≥8 years) were included. Four-point Dixon MRI scans of the right upper arm were performed at baseline and at the 12-, 18-, or 24-month follow-up. Elbow flexor FFs were determined from 5 central slices. Loss of hand-to-mouth movement was determined at study visits and by phone calls every 4 months. FFs were fitted to a sigmoidal curve by use of a mixed model with random slope to predict individual trajectories. The added predictive value of elbow flexor FF to age at loss of hand-to-mouth movement was calculated from a Cox model with the predicted FF as a time-varying covariate, yielding a hazard ratio.

**Results:**

Forty-eight MRIs of 20 patients with DMD were included. The hazard ratio of a percent-point increase in elbow flexor FF for the time to loss of hand-to-mouth movement was 1.12 (95% confidence interval 1.04–1.21; *p* = 0.002). This corresponded to a 3.13-fold increase in the instantaneous risk of loss of hand-to-mouth movement in patients with a 10–percent points higher elbow flexor FF at any age.

**Discussion:**

In this prospective study, elbow flexor FF predicted loss of hand-to-mouth movement independently of age. qMRI-measured elbow flexor FF can be used as a surrogate endpoint or stratification tool for clinical trials in nonambulant patients with DMD.

**Classification of Evidence:**

This study provides Class II evidence that qMRI FF of elbow flexor muscles in patients with DMD predicts loss of hand-to-mouth movement independently of age.

Duchenne muscular dystrophy (DMD) is characterized by muscle weakness in a proximal-to-distal gradient. Independent ambulation is generally lost in the early teens and occurs years before loss of hand-to-mouth movement (LoHM).^1^ While the first drugs for ambulant DMD have received conditional approval, results cannot be extrapolated to later stages of the disease due to progressive and irreversible replacement of muscle by fat and fibrosis, causing a reduction in target tissue.^2,3^ Conducting clinical trials in DMD is challenging and may be facilitated by objective biomarkers that can be used for stratification or as a surrogate endpoint. Quantitative MRI (qMRI) fat fraction (FF) of the vastus lateralis muscle has been shown to predict loss of ambulation in DMD.^4,5^ This predictive value must be additive to age because any parameter that consistently changes over time will correlate to a decline in function in a progressive disease. Upper arm qMRI FF increases over time and correlates with function cross-sectionally.^6,7^ We studied the additive predictive value of elbow flexor FF (FF_EF_) for LoHM to age in a prospective study in nonambulant patients with DMD.

## Methods

We included male nonambulant patients with genetically confirmed DMD who were ≥8 years of age between March 2018 and July 2019. Patients were recruited from the Dutch Dystrophinopathy Database^8^ and through Dutch outpatient clinics and patient organizations. Exclusion criteria were MRI contraindications (e.g., spinal fusion, daytime respiratory support, or inability to lie still for 45 minutes), exposure to an investigational drug ≤6 months before participation, and recent (≤6 months) upper extremity surgery or trauma. One hundred twenty-two eligible patients were approached for participation; details on this recruitment have been reported previously.^9^ Patients visited the Leiden University Medical Center (LUMC) for a half-day of assessments at baseline and 12 and 18 months. Due to the coronavirus disease 2019 (COVID-19) pandemic, some follow-up visits were postponed from 12 to 18 months and from 18 to 24 months or missed. Telephone calls every 4 months were used to evaluate LoHM.

### Standard Protocol Approvals, Registrations, and Patient Consents

The medical ethics committee of the LUMC approved the study, and we obtained written informed consent from patients and legal representatives. The study was registered on ToetsingOnline (NL63133.058.17).

### MRI Acquisition and Analysis

**Figure 1:**
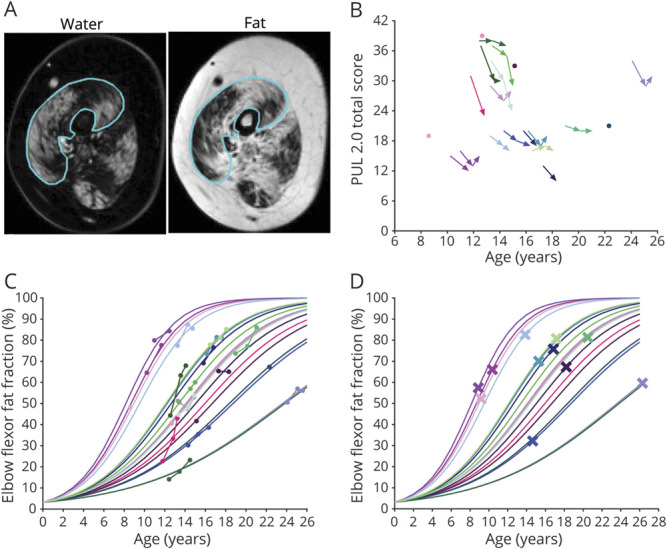
Longitudinal Clinical and FF_EF_ Data (A) Example of a region of interest drawn on the elbow flexor muscles (line) is shown on a water image (left) and corresponding fat image (right). (B) Longitudinal performance of the upper limb (PUL) 2.0 total scores (maximum 42 points) are plotted vs age. PUL total scores decrease with age, but there is a large variation in scores between patients with Duchenne muscular dystrophy of similar ages. (C) Elbow flexor fat fraction (FF_EF_) results that were acquired are plotted vs age, as well as FF_EF_ results that were predicted with a logit transformation, linear (mixed) model, and logistic transformation. Patients with higher FF_EF_ results at younger ages or faster FF_EF_ increases had steeper predicted FF_EF_ slopes. (D) Predicted FF_EF_ results are plotted vs age, and predicted FF_EF_ at age at loss of hand-to-mouth movement is shown with a cross. Colors used in panels B–D are unique for each participant.

Four-point Dixon scans were acquired of the right upper arm on a 3T MRI scanner (Ingenia, Philips Healthcare, Best, the Netherlands) with 2 circular 15-cm coils. Participants were positioned on the right side with the right shoulder and elbow in 90° flexion because pilot experiments suggested this to be the most comfortable position and this position placed the upper arm muscles more toward the center of the MRI scanner. If this was uncomfortable, a supine position was chosen. Dixon scans were acquired with 33 slices and a voxel size of 1 × 1 × 10 mm (repetition time 310 milliseconds, first echo 4.40 milliseconds, echo spacing 0.76 milliseconds, flip angle 20°) and aligned perpendicular to the humerus bone. Dixon water and fat images were generated with in-house developed software (Matlab 2016a, MathWorks, Natick, MA) with a 6-peak lipid spectrum; B0 maps were calculated from the phase data of the first and last echoes. Regions of interest (ROIs) were drawn on the muscle border of the elbow flexors (biceps and brachialis muscles) by 1 reviewer (K.J.N.) on 5 contiguous slices around a central slice (Figure 1A) using the mipav application (Medical Image Processing, Analysis, and Visualization, NIH, Center for Information Technology, version 7.4.0). The central slice was located at 40% distance from the elbow based on the length of the humerus bone. The same reviewer performed quality control of elbow flexor ROIs in which scans with ROIs that contained artifacts or insufficient signal were excluded. ROIs from different time points on similar slices in the same participant were also compared by this reviewer and adjusted in case of discrepancies. ROIs were eroded by 2 voxels, and FF_EF_ was calculated as a weighted mean value by averaging elbow flexor voxels of all eroded ROIs from the reconstructed fat and water images and correcting for partial saturation due to T1 effects:
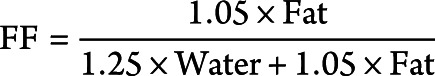


### Clinical Assessments and Endpoint

Performance of the upper limb (PUL) 2.0 was assessed for the right arm at all visits. LoHM was defined as the inability to move a filled glass independently to the mouth with the right hand and allowing support of the elbow on a table, similar to the PUL hand-to-mouth item in which a 200-g weight is used. Age at LoHM was prospectively established to a month's precision. If LoHM had occurred before baseline, month and year were established retrospectively through a detailed interview and clinical documentation.

### Statistical Analysis

The difference in FF_EF_ between baseline and the 12-month follow-up was assessed with the Wilcoxon signed-rank test. The additive predictive value of FF_EF_ to age at LoHM was calculated with a Cox proportional hazards model as described previously.^4^ For this, we applied a logit transformation to FF_EF_ to allow use of standard statistical methods that rely on a gaussian distribution. A linear (mixed) model was fitted to the transformed data with age as the only covariate and a random slope per individual. The fitted lines were transformed back to the original scale with a logistic transformation, after which individual FF_EF_ trajectories were predicted at any time. The Cox proportional hazards model was fitted with the predicted FF_EF_ as a time-varying covariate, yielding a hazard ratio (Wald test; *p* < 0.05).

The primary research question of this study was the following: does FF_EF_ have additive predictive value to age at LoHM in nonambulant patients with DMD? The level of evidence was assigned as Class II during the review process.

### Data Availability

Anonymized data and analysis software can be made available to qualified investigators.

## Results

Twenty-two patients with DMD participated, but 2 patients refused the MRI. One patient switched to a medication trial after baseline; 1 patient discontinued after 12 months of follow-up because of traveling distance; and 8 visits were canceled due to COVID-19 restrictions. One 12-month follow-up scan was excluded due to insufficient signal. Forty-eight MRIs of 20 patients with DMD were included: 12 patients had 3 MRIs; 4 patients had 2; and 4 patients had 1. All patients used glucocorticoids, but 1 patient had temporarily ceased treatment 6 weeks before baseline due to weight gain. Patients' characteristics at baseline and FF_EF_ results and PUL scores at different time points are presented in the Table. LoHM had occurred before baseline in 2 patients and during the study in 9. Median decline in PUL total score over 12 months was 3 points (n = 15, range −1 to 8). Median decline in PUL elbow domain score was 2 points (range 0–4; Figure 1B). There was a significant mean annual increase in FF_FE_ of 5.9 ± 5.4% (*p* < 0.01).

**Table:**
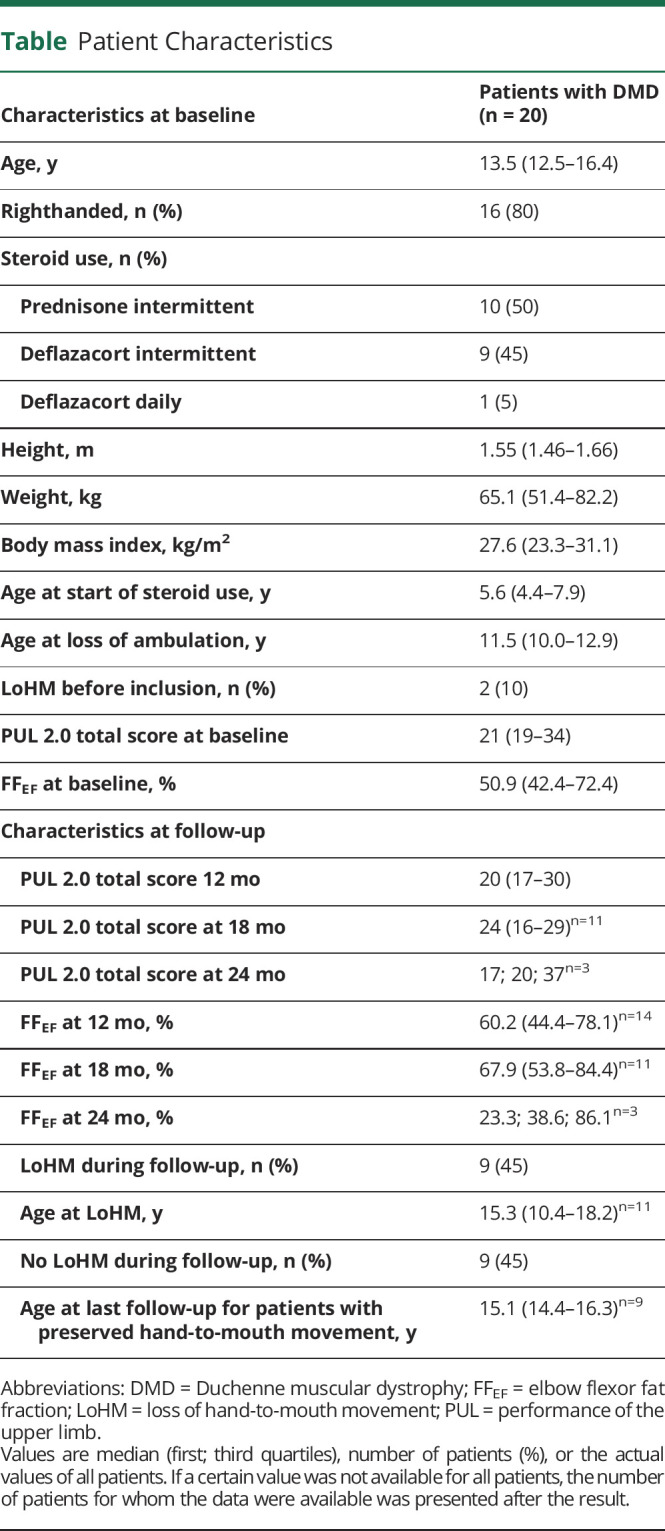
Patient Characteristics

### Relation Between Hand-to-Mouth Movement and FF_EF_

**Figure 2:**
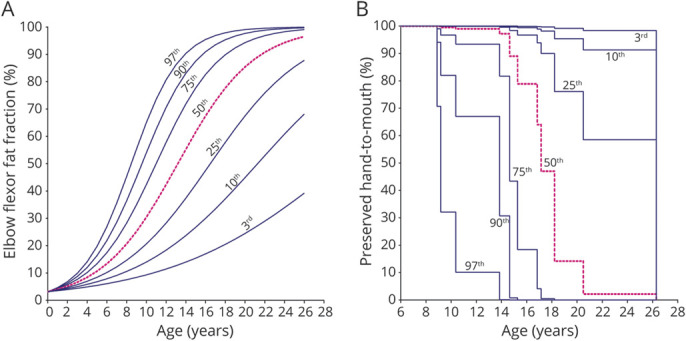
Elbow Flexor Fat Fraction and Preserved Hand-to-Mouth Movement vs Age In (A) we generated an FFEF growth chart with a 3rd, 10th, 25th, 50th, 75th, 90th and 97th percentile curve from the predicted FFEF data. (B) Using the resulting hazard ratio, we transformed the predicted FFEF growth curves to a 3rd, 10th, 25th, 50th, 75th, 90th and 97th percentile survival curve for preserved hand-to-mouth movement. A patient on the 3rd percentile in the FFEF growth chart is also on the 3rd percentile in the survival chart.

Acquired and predicted FF_EF_ data and predicted FF_EF_ at age at LoHM are shown in Figure 1, C and D, respectively. The hazard ratio of a percent-point increase in FF_EF_ for the time to LoHM was 1.12 (log hazard ratio 0.11, 95% confidence interval 1.04–1.21; *p* = 0.002). This hazard ratio corresponds to a 3.13-fold increase in the instantaneous risk of LoHM in patients with a 10–percent points higher FF_EF_ at any age. An FF_EF_ growth chart (Figure 2A) and survival chart for preserved hand-to-mouth movement (Figure 2B) illustrate relationships between percentile FF_EF_ curves and LoHM trajectories.

## Discussion

In this prospective study, we show that FF_EF_ predicts LoHM in nonambulant patients with DMD on top of age. This added predictive value is essential because parameters that consistently change over time will always correlate with functional tests in a progressive disease.

Previous studies demonstrated that qMRI muscle FF increases over time and correlates with function cross-sectionally in DMD.^6,7,10,11^ However, any outcome measure that consistently changes over time will correlate to declining measures of function in a progressive disease. Two previous studies demonstrated the added predictive value of vastus lateralis FF on top of age on the clinical outcome loss of ambulation and thus showed for the first time that muscle FF adds to the assessment of disease severity.^4,5^

FF_EF_ increased according to a sigmoidal curve, similar to the vastus lateralis FF in ambulant patients.^4,5^ The hazard ratio of 1.12 was comparable to that of the vastus lateralis FF for the time to loss of ambulation.^4^ These data thus support the use of qMRI FF as an objective biomarker in different stages of the disease. Predicted FF curves can be used for stratification in clinical trials or as a surrogate endpoint, limiting sample size and duration.

The rate of change in FF, for instance, 1-year change, could be used as a biomarker in trials in which the therapeutic effect over that period of time can be compared to placebo or another therapy, and the power calculation could be based on the more or less “linear” middle part of the FF curve because that is where the fastest change is expected to happen. This will require stratification of the cohort with respect to baseline FF, as is now commonly done for functional tests.^10^

We assessed the timing of reaching the clinical endpoint via regular phone calls between clinical assessments. In our experience, patients and caregivers are able to define such important endpoints within a month's precision. This increases the power of our survival analyses compared to standard natural history studies in which clinical assessments are performed at 6- or 12-month intervals only. It reduced the burden for participants and allowed continuation of the protocol despite COVID-19–related restrictions. The importance of hand-to-mouth movement is stressed by its incorporation into the widely used Brooke upper extremity rating scale, the PUL, and the DMD Upper Limb Patient Reported Outcome Measure, in which patients and families confirmed its clinical relevance.^12,13^

Limitations of this study include the small sample size, which did not allow modeling of the intercept of the FF_EF_ curves. Restrictions due to the COVID-19 pandemic also led to some missing data. It is important to replicate results in other cohorts with different steroid regimens.

FF_EF_ predicted LoHM independently of age in nonambulant patients with DMD. This establishes qMRI FF as a biomarker in DMD and potentially facilitates the design of clinical trials, through either stratification or use as a surrogate endpoint.

## Glossary

COVID-19: coronavirus disease 2019
DMD: Duchenne muscular dystrophy
FF: fat fraction
LoHM: loss of hand-to-mouth movement
LUMC: Leiden University Medical Center
PUL: performance of the upper limb
qMRI: quantitative MRI
ROI: regions of interest

## References

[1] McDonald CM, Henricson EK, Abresch RT, et al. Long-term effects of glucocorticoids on function, quality of life, and survival in patients with Duchenne muscular dystrophy: a prospective cohort study. Lancet. 2018;391(10119):451-461. 29174484 10.1016/S0140-6736(17)32160-8

[2] European Medicines Agency (EMA). CHMP guideline on the clinical investigation of medicinal products for the treatment of Duchenne and Becker muscular dystrophy. Accessed December 18, 2020. Available at: www.ema.europa.eu/docs/en_GB/document_library/Scientific_guideline/2015/12/WC500199239.pdf.

[3] Verhaart IEC, Aartsma-Rus A. Therapeutic developments for Duchenne muscular dystrophy. Nat Rev Neurol. 2019;15(7):373-386. 31147635 10.1038/s41582-019-0203-3

[4] Naarding KJ, Reyngoudt H, van Zwet EW, et al. MRI vastus lateralis fat fraction predicts loss of ambulation in Duchenne muscular dystrophy. Neurology. 2020;94(13):e1386-e1394. 31937624 10.1212/WNL.0000000000008939 PMC7274919

[5] Rooney WD, Berlow YA, Triplett WT, et al. Modeling disease trajectory in Duchenne muscular dystrophy. Neurology. 2020;94(15):e1622-e1633. 32184340 10.1212/WNL.0000000000009244 PMC7251517

[6] Forbes SC, Arora H, Willcocks RJ, et al. Upper and lower extremities in Duchenne muscular dystrophy evaluated with quantitative MRI and proton MR spectroscopy in a multicenter cohort. Radiology. 2020;295(3):616-625. 32286193 10.1148/radiol.2020192210 PMC7263287

[7] Wood CL, Hollingsworth KG, Hughes E, et al. Pubertal induction in adolescents with DMD is associated with high satisfaction, gonadotropin release and increased muscle contractile surface area. Eur J Endocrinol. 2020;184(1):67-79. 10.1530/EJE-20-0709 33112266

[8] van den Bergen JC, Ginjaar HB, van Essen AJ, et al. Forty-five years of Duchenne muscular dystrophy in the Netherlands. J Neuromuscul Dis. 2014;1(1):99-109. 27858664

[9] Naarding KJ, Doorenweerd N, Koeks Z, et al. Decision-making and selection bias in four observational studies on Duchenne and Becker muscular dystrophy. J Neuromuscul Dis. 2020;7(4):433-442. 32925089 10.3233/JND-200541 PMC7902964

[10] Barnard AM, Willcocks RJ, Triplett WT, et al. MR biomarkers predict clinical function in Duchenne muscular dystrophy. Neurology. 2020;94(9):e897-e909. 32024675 10.1212/WNL.0000000000009012 PMC7238941

[11] Barnard AM, Willcocks RJ, Finanger EL, et al. Skeletal muscle magnetic resonance biomarkers correlate with function and sentinel events in Duchenne muscular dystrophy. PLoS One. 2018;13(3):e0194283. 29554116 10.1371/journal.pone.0194283 PMC5858773

[12] Mayhew A, Mazzone ES, Eagle M, et al. Development of the performance of the upper limb module for Duchenne muscular dystrophy. Dev Med Child Neurol. 2013;55(11):1038–1045. 23902233 10.1111/dmcn.12213

[13] Klingels K, Mayhew AG, Mazzone ES, et al. Development of a patient-reported outcome measure for upper limb function in Duchenne muscular dystrophy: DMD Upper Limb PROM. Dev Med Child Neurol. 2017;59(2):224-231. 27671699 10.1111/dmcn.13277

